# The Voluntary Hospitals and the Government: the Gist of the Matter

**Published:** 1912-12-21

**Authors:** 


### THE VOLUNTARY HOSPITALS AND THE GOVERNMENT.

### The Gist of the Matter.

It will be seen from the following official communication we have received from the Secretary to the National Health Insurance Commissioners that the Chancellor of the Exchequer has in fact committed the Government to the statement that insured persons will need as much as ever the aid of the voluntary hospitals in order to obtain the treatment that is given in the in-patient departments, and to a substantial extent in the out-patient departments too. Hospital opinion has immediately recognised that this speech shows clearly that the Chancellor has not, in fact, any apprehension of the dangers to the voluntary hospitals, to the financial strength of approved societies, and to the health of the working community of these islands which must result from the National Insurance Act as it stands, unless the light of knowledge on these dangers can be brought to bear on the counsels of the Government. The National Insurance Act threatens the majority of voluntary hospitals with imminent danger from the possible loss of a large proportion of their present revenues, whilst experience of past legislation proves it will bring to every voluntary hospital of importance possibly 50 per cent. more patients than heretofore, all of whom will stand in pressing need of the in-patient treatment or of the special medical and surgical skill which the Chancellor declares insured persons must seek at the voluntary hospitals if they are to secure its advantages. The will to do all the expensive work which the Chancellor declares must devolve upon voluntary hospitals is possessed by hospital managers in abundance; but where is the money to come from to defray the cost of such treatment? As Mr. E. W. Morris states in the *Daily Telegraph* of the 18th inst., "There is no quarrel between the voluntary hospitals and the Government; it is between the Government and the poor who may ask, 'Where does the 9d. for 4d. come in?'" This speech of Mr. Lloyd George will bring sorrow and painful anxiety into many poor homes this Christmas. We believe the utterances in question to be based upon an entire absence of practical knowledge of the real issues raised by the National Insurance Act. We hope the poor may find consolation in our belief that, when these evils and dangers are brought home to the knowledge and understanding of the Chancellor, means will be found to protect the poor from the many evils which must otherwise arise from the policy embodied in the following official report issued by the National Health Insurance Commissioners at the instance of Mr. Lloyd George.

### The Official Report.

An interview took place on Tuesday afternoon between the Chairmen of some of the larger London Hospitals having medical schools and the Chancellor of the Exchequer. Various points were discussed regarding the effect of the Insurance Act in relation to voluntary hospitals. The Chancellor explained that the main work of the hospitals is not touched by the Act, inasmuch as the treatment afforded to insured persons under the Act is such treatment as can properly be given by a general practitioner of ordinary competence and skill, whereas this is just the kind of treatment that a hospital does not exist to give. Thus insured persons need as much as ever the aid of hospitals in order to obtain the treatment that is given in the in-patient departments and to a substantial extent that given in the out-patient departments—*e.g.*, all that for which special medical or surgical skill is required. Thus hospitals would only be carrying out their proper duty in continuing to give this treatment. Those subscriptions, donations, and legacies on which the hospitals have depended would be needed as much as hitherto, and they would have the same claim on the support of the benevolent.

The Chancellor said that the question of hospitals had been by no means overlooked in planning the Government Insurance scheme, but that it had been deemed wiser to endeavour to avoid any steps that would imperil the voluntary nature of the great hospitals of this country. This could not have been avoided if hospital work had been brought into the scheme of the Act, since a subsidy from public funds would necessarily be followed by some degree of public control. He understood that the hospital authorities were greatly desirous of continuing the voluntary system unimpaired; and, if the position of things under the Act regarding hospitals is properly understood, it will be seen that no fears need be entertained on this score from the Act. Certain special points were also submitted for the Chancellor's consideration regarding the effect of the Act upon the financial position of the hospitals, to which he promised consideration.

The Chairmen of Hospitals present were: Charing Cross Hospital (Mr. Robert Duff), Guy's Hospital (Lord Goschen), King's College Hospital (the Hon. W. F. D. Smith), London Hospital (the Hon. Sydney Holland), Middlesex Hospital (Prince Alexander of Teck), Royal Free Hospital (the Earl of Sandwich), St. Bartholomew's Hospital (Lord Sandhurst), St. George's Hospital (Mr. William West), St. Mary's Hospital (Mr. Austen Leigh), St. Thomas's Hospital (Mr. Wainwright), University Hospital (Captain the Hon. E. Dawson), Westminster Hospital (Sir John Wolfe Barry).

